# Synthesis of Tryptoline-3-Carboxylic Acid Derivatives A Novel Antidiabetic Agent

**DOI:** 10.4103/0975-1483.80302

**Published:** 2011

**Authors:** AN Choudhary, MS Kohli, A Kumar, A Joshi

**Affiliations:** *Department of Pharmaceutical Sciences, Bhimtal Campus, Bhimtal, Kumaun University, Nainital 263136, Uttrakhand, India*; 1 *Ranbaxy Research Ltd., Gurgoan, Haryana-122002, India*

**Keywords:** Antidiabetic agents, 2-(N-hydroxymethyl amino)-indol-3-yl-propanoic acid, 1,2,3,4-tetrahydro-9Hpyrido [3,4-b] indole-3-carboxylic acid, Peroxisome proliferator-activated receptorg (PPAR-γ) agonists

## Abstract

The compounds, 2-(methylsulfonyl)-1,2,3,4-tetrahydro-9*H*-pyrido[3,4-b]indole-3-carboxylic acid (DM3), 2-(phenylsulfonyl)-1,2,3,4-tetrahydro-9*H*-pyrido[3,4-b]indole-3-carboxylic acid (DM_4_), and 2-(*p*-toluenesulfonyl)-1,2,3,4-tetrahydro-9*H*-pyrido[3,4-b]indole-3-carboxylic acid (DM_5_) were synthesized by coupling of 1,2,3,4-tetrahydro-9*H*-pyrido[3,4-b]indole-3-carboxylic acid (DM_2_) with methanesulfonyl chloride, benzenesulfonyl chloride, and toluenesulfonyl chloride, which in turn, was synthesized by dissolving dilute aqueous ammonia with 2-(N-hydroxy methyl amino)-indol-3-yl-propanoic acid (DM_1_) which is the reaction product of l-tryptophan and formalin. All the intermediates and title compounds were characterized by physical, chemical, analytical, and spectral data. All the title compounds have been screened for *in vivo* antidiabetic activity in streptozotocin-induced diabetic rats, and serum glucose was estimated spectrophotometrically at 505 nm by glucose oxidase/peroxidase method. Compound DM_5_ showed potent antidiabetic activity.

## INTRODUCTION

Diabetes mellitus is a major health concern, especially in the urban world.[1] Over 90% of the diabetes mellitus patients are type-2 patients.[2] Type-2 diabetes mellitus is characterized by insulin resistance and cardiovascular dysmetabolic syndrome.[3] The conventional therapy of type-2 diabetes mellitus has not been satisfactory as it is not successful in treating associated cardiovascular risk factors, which is the main cause of morbidity.[4] The current trend is therefore, to make therapy better by choosing appropriate combination of available drugs. A parallel search for newer drugs is also being made.

Thiazolidine-2,4-diones are the class of oral hypoglycemic agents which increase insulin sensitivity at target tissues such as liver and skeletal muscles. In addition, it also improves the markers of cardiovascular risk factors by decreasing the free fatty acids and altering the lipoprotein metabolism.[5] Thiazolidine-2,4-diones act on peroxisome proliferators activating receptor-γ (PPAR-γ) receptors which regulate the gene expression mainly in the adipose tissues.[6–8] Rosiglitazone and pioglitazone molecules from the class of thioglitazone available in the market are showing severe adverse effects.[9,10]

A survey of literature revealed that replacement of the thiazolidine-2,4-dione ring by various acidic groups such as α-heteroatom-substituted carboxylic acids and α-carbon-substituted carboxylic acids can results in qualitative and quantitative changes in the activity,[11–13] which prompted us to undertake the synthesis of various new derivatives with the aim of having improved activity and less toxicity.

## MATERIALS AND METHODS

The melting points were recorded in open sulphuric acid or oil bath using thermometer and were uncorrected. IR spectra were recorded using Hitachi 270-30 infrared and Bruker Vector 22 spectrophotometers using KBr pellet techniques. ^1^H-NMR spectrum was recorded using DMSO-d _6_ on Bruker Avance DPX-200 at 300 MHz, and their chemical shifts are recorded in δ (parts per million) units with respect to tetramethyl silane (TMS) as an internal standard. Atmospheric pressure ionization (API) mass spectra were recorded on Bruker Ion Trap Esquire 3000 spectrometer with the ionization potential 3000 V. Progress of the reactions was monitored using TLC, performed on aluminum plates precoated with silica gel-G, using chloroform–methanol (92:8) as the solvent systems, and the spots were visualized by exposure to iodine vapors. The physical constants of the title compounds are reported in Table 1

**Table 1:** Physical constants of intermediates and title compounds

Compound code	Molecular formula	Molecular weight	Yield, %	Melting point (ºC)	Solvent system chloroform:methanol	*R*_f_ values
DM_1_	C_12_H_12_N_2_O_3_	234.32	75	230–231	92:8	0.50
DM_2_	C_12_H_12_N_2_O_2_	216.23	80	312–313	92:8	0.72
DM_3_	C_12_H_14_N_2_O_4_S	294.32	70	240–242	92:8	0.62
DM_4_	C_18_H_16_N_2_O_4_S	356.39	78	262–263	92:8	0.69
DM_5_	C_19_H_18_N_2_O_4_S	370.42	74	274–275	92:8	0.67

### Procedure for the preparation of 2-(*N*-hydroxymethyl amino)-indol-3-yl-propanoic acid (DM_1_)

In a 250-mL conical flask, 5 g (0.0245 M) of l-tryptophan was dissolved in 120 mL of water. To this, 20 mL formalin was added and the mixture was incubated at 38°C for 6 h. The colorless, crystalline, but rather granular solid that had then separated was collected, washed with cold water, and dried in vacuum. The crude product was recrystallized from hot water.

**IR (KBr) (cm^-1^):** 3600–3400 (m) broad O–H stretching, 3300 (m) N–H stretching, 3099 (m) aromatic C–H stretching (asymmetric), 3056 (m) aromatic C–H stretching (symmetric), 2989 (m) aliphatic C–H stretching (asymmetric), 2852 (m) aliphatic C–H stretching (symmetric), 1680 (s) carbonyl stretching 1620, 1552, 1447 (m) skeleton in plane vibrations C=C, 1408–1425 (s) C=C and C=N ring stretching, 750 (m) N–H wagging, 741 (s) out of plane C=C bending.

**^1^H-NMR (DMSO-d^6^, 300 MHz), δ (ppm):** 7.36–7.45 (m, 4H, ArH), 7.10 (s, 1H, α proton of indole ring), 3.70–3.80 (s, 1H, aromatic N-H, D_2_O exchangeable), 2.80 {d, 2H, –CH_2_ (*J*_vic_ = 7.2)}, 3.88 {t, 1H, –CH (*J*_vic_ = 7.2)}, 11.00 (s, 1H, COOH), 2.10 (s, 1H, NH, D_2_O exchangeable), 4.60 (s, 2H, –CH_2_), 4.10 (s, 1H, OH).

**Mass spectra:** Molecular ion peak M *at m/e* 234 (23.8%), [M–(OH)]^+^and [M–(H_2_O)]^+^shows peak at *m/e* 217 (32.4%), 216 (100%). The other important ions are at m/e 190 (10.5%), 158 (8.4%), 157 (11.8%), 156 (15.3), 144 (10.8%), 117 (18.6%), and 91 (8.5%).

**Procedure for the preparation of 1,2,3,4-tetrahydro- 9*H*-pyrido [3,4-b] indole-3-carboxylic acid (DM_2_):** In a 250-mL conical flask, 150 mL of dilute aqueous ammonia was placed and 5 g (0.0214 M) of α-hydroxymethylamino-α-3-indolylpropionic acid (DM_1_) was added and dissolved. Then, this solution was refluxed for 3 h and concentrated to a small volume. The white-to-cream colored crystals obtained were given charcoal treatment and filtered by suction, washed with cold water and dried at 80°C. The product was obtained in colorless leaflets and was purified by recrystallization from hot water.

**IR (KBr) (cm^-1^):** 3650–3300 (m) broad O–H stretching, 3350 (m) N–H stretching, 3100 (m) aromatic C–H stretching (asymmetric), 3056 (m) aromatic C–H stretching (symmetric), 2990 (m) aliphatic C–H stretching (asymmetric), 2850 (m) aliphatic C–H stretching (symmetric), 1690 (s) carbonyl stretching 1600, 1582, 1450 (m) skeleton in plane vibrations C=C, 1410–1430(s) C=C and C=N ring stretching, 760 (m) N–H wagging, 752 (s) out of plane C=C bending.

**^1^H-NMR (DMSO-d^6^, 300 MHz), δ (ppm):** 7.10–7.56 (m, 4H, ArH), 3.70–3.80 (s, 1H, aromatic N–H, D_2_O exchangeable), 2.80 {d, 2H, –CH_2_(*J*_vic_ = 7.2)}, 3.88 {t, 1H, CH (*J_vic_* = 7.2)}, 11.00 (s, 1H, COOH), 2.10 (s, 1H, NH of piperidine ring, D_2_O exchangeable), 4.10 (s, 2H, CH_2_).

**Mass spectra:** Molecular ion peak M at *m/e* 216 (100%), [M–(OH)] ^+^and [M–(C_2_H_2_)] ^+^shows peak at *m/e* 199 (18.4%), 190 (27.2%). The other important ions are at *m/e* 177 (19.7%), 144 (14.3%), 118 (5.8%), 117 (20.5%), 91 (6.2%), 91 (5.8%).

### Procedure for the preparation of 2-(methylsulfonyl)- 1,2,3,4-tetrahydro-9*H*-pyrido [3,4-b]indole-3- carboxylic acid (DM_3_)

Treated 3 g (0.01389 M) of the amine (DM_2_) with 50 mL of 10% sodium hydroxide solution and 2 mL (3.5 g, 0.0303 M) of methanesulfonyl chloride was added in small proportions. The reaction mixture was stirred vigorously for 30 min and warmed it slightly with continued shaking. When the odor of methanesulfonyl chloride was dissipated, the mixture was cooled in an ice bath. As oil separated, crystallization was induced by rubbing the inner wall of the beaker with the glass rod in an ice bath. Any solid produced should be separated by filtration; and the crude product was recrystallized from dilute ethanol to give pure DM_3_ as light brown crystals.

**IR (KBr)(cm^-1^) :** 3600–3400 (Broad O–H stretching), 3300 (N–H stretching), 3099 (aromatic C–H stretching asymmetric), 3056 (aromatic C–H stretching symmetric), 2989 (aliphatic C–H stretching asymmetric), 2852 (aliphatic C–H stretching symmetric), 1680 (carbonyl stretching), 1620, 1552, 1447 (skeleton in plane vibrations C=C), 748 (N–H wagging), 741 (s) out of plane C=C bending, 1330 (s) SO_2_ stretching asymmetric, 1120 (s) SO_2_ stretching symmetric, 560 (s) SO_2_ rocking, 530 (s) SO_2_ scissoring.

**^1^H-NMR (DMSO-d^6^, 300 MHz), δ (ppm):** 7.10–7.40 (m, 4H, ArH), 3.70–3.80 (s, 1H, aromatic N–H, D_2_O exchangeable), 2.80 {d, 2H, –CH_2_ *J*_vic_ = 7.2)}, 3.88 {t, 1H, –CH *J*_vic_ = 7.2)}, 11.00 (s, 1H, COOH), 2.90 (s, 3H, –SO_2_CH_3_), 3.80–3.85 (m, 2H, –CH_2_).

**Mass spectra:** Molecular ion peak M at *m/e* 294 (43.9%), [M–(OH)] ^+^shows peak at *m/e* 277 (26.2%). The other important ions are at *m/e* 249 (10.8%), 216 (100%), 144 (12.2%), 118 (6.4%), 117 (20.5%), 107 (6.2%), 91 (16.5%), 79 (8.4%).

### Procedure for the preparation of 2-(phenylsulfonyl)- 1,2,3,4-tetrahydro-9*H*-pyrido [3,4-b] indole-3- carboxylic acid (DM_4_)

To a 100-mL round bottom flask was added 3 g (0.01389 M) of amine (DM_2_), 20 mL of dry pyridine, and 2 mL (2.75 g, 0.01559 M) of benzene sulfonylchloride. The reaction mixture was refluxed for 2 h on heating mantle and poured into crushed ice to give a light-brownish solid. As oil separated, crystallization was induced by rubbing the inner wall of the beaker with the glass rod in an ice bath. Any solid produced should be separated by filtration, and the crude product was recrystallized from dilute ethanol to give pure DM_4_ as light brown crystals.

**IR (KBr) (cm ^-1^) :** 3600–3400 (m) (broad O–H stretching), 3300 (m) (N–H stretching), 3099 (m) (aromatic C–H stretching asymmetric), 3056 (m) (aromatic C–H stretching symmetric), 2989 (m) (aliphatic C–H stretching asymmetric), 2852 (m) (aliphatic C–H stretching symmetric), 1680 (s) (carbonyl stretching), 1620, 1552, 1447 (m) (skeleton in plane vibrations –C=C), 1408–1425 (s) (C=C and C=N ring stretching), 750 (N–H wagging), 741 (s) out of plane C=C bending, 1338 (s) SO_2_ stretching asymmetric, 1118 (s) SO_2_ stretching symmetric, 565 (s) SO_2_ rocking, 532 (s) SO_2_ scissoring.

**H^1^ NMR (DMSO-d^6^, 300 MHz), δ (ppm):** 7.05–7.10 (m, 4H, ArH), 3.70–3.80 (s, 1H, aromatic N–H, D_2_O exchangeable), 2.80 {d, 2H, –CH_2_ (*J*_vic_ = 7.2)}, 3.86 {t, 1H, –CH (*J*_vic_ = 7.2)}, 11.00 (s, 1H, COOH), 7.95 (d, 2H, o-protons of ArH with respect to SO_2_ group), 7.30–7.50 (m, 3H, ArH with respect to SO_2_), 4.55–4.60 (m, 2H, –CH_2_).

**Mass spectra:** Molecular ion peak M at *m/e* 356 (43.9%), [M–(OH)] ^+^at *m/e* 339 (18.8%). The other important ions are at *m/e* 330 (21.2%), 311 (13.5%), 216 (100%), 169 (16.44%), 144 (20.54%), 141 (6.5%), 118 (22.4%), 117 (23.7%), 91 (8.5%), 77 (9.4%), 51 (6.2%)

### Procedure for the preparation of 2-(p-toluenesulfonyl)- 1,2,3,4-tetrahydro-9*H*-pyrido[3,4-b]indole-3- carboxylic acid (DM_5_)

Treated 3g (0.01389 M) of the amine (DM_2_) with 50 mL of 10% sodium hydroxide solution and 3 g (0.01574 M) of *p*-toluenesulfonyl chloride was added in small proportions. The reaction mixture was stirred vigorously for 30 min and warmed it slightly and shaking was continued. When the odor of p-toluenesulfonyl chloride was dissipated, the mixture was cooled in an ice bath. As oil started separating, crystallization was induced by rubbing the inner wall of the beaker with the glass rod in an ice bath. Any solid produced should be separated by filtration, and the crude product was recrystallized from dilute ethanol to give pure DM_5_ as light brown crystals.

**IR (KBr) cm^-1^:** 3600–3400 (m) (broad O–H stretching), 3300 (m) (N–H stretching), 3099 (m) (aromatic C–H stretching asymmetric), 3056 (m) (aromatic C–H stretching symmetric), 2989 (m) (aliphatic C–H stretching asymmetric), 2852 (m) (aliphatic C–H stretching symmetric), 1680 (s) (carbonyl stretching), 1620, 1552, 1447 (m) (skeleton in plane vibrations C=C), 1408–1425 (s) (C=C and C=N ring stretching), 748 (m) (N–H wagging), 740 (s) out of plane C=C bending, 1328 (s) SO_2_ stretching asymmetric, 1122 (s) SO_2_ stretching symmetric, 560 (s) SO_2_ rocking, 532 (s) SO_2_ scissoring.

**^1^H-NMR (DMSO-d ^6^, 300 MHz), δ (ppm):** 7.05–7.10 (m, 4H, ArH), 3.70–3.80 (s, 1H, aromatic N–H, D_2_O exchangeable), 2.80 {d, 2H, –CH_2_ *(J*_vic_ = 7.2)}, 3.88 {t, 1H, –CH *(J*_vic_ = 7.2)}, 11.00 (s, 1H, COOH), 7.80 (d, 2H, *o*-protons of ArH with respect to SO_2_ group), 7.30–7.45 (m, 2H, m-protons of ArH with respect to SO_2_ group), 2.45 (s, 3H, –CH_3_), 4.55–4.62 (m, 2H, –CH_2_).

**Mass spectra :** Molecular ion peak M at *m/e* 370 (44.2%) and [M–(OH)] ^+^ shows peak at *m/e* 353 (11.9%). The other important ions are at *m/e* 330 (21.4%), 325 (13.9%), 216 (100%), 183 (6.44%), 155 (20.5%), 144 (14.2%), 118 (9.2%), 117 (22.88%), 91 (8.5%), 91 (9.4%).

### Evaluation of antidiabetic activity

The synthesized compounds were screened for *in vivo* antidiabetic activity in streptozotocin-induced diabetic rats. After administration of standard drug (pioglitazone) and synthesized compounds (DM_1–5_), serum glucose was estimated spectrophotometrically at 505 nm by glucose oxidase/peroxidase method using a commercially available kit (Span Diagnostic Ltd, Surat, India).

Concentration of glucose (mg/dL) = Optical density of test/Optical density of std. × 100.

The results are summarized in Table 2.

**Table 2:** Antidiabetic activity of synthesized compounds against streptozotocin-induced diabetes in rats

Groups	Blood glucose concentration (mg/dL)
DM_1_	176
DM_2_	179
DM_3_	154
DM_4_	167
DM_5_	145
Control	118
Streptozotocin treated	187
Pioglitazone treated	135

## RESULTS AND DISCUSSION

**Scheme 1:**
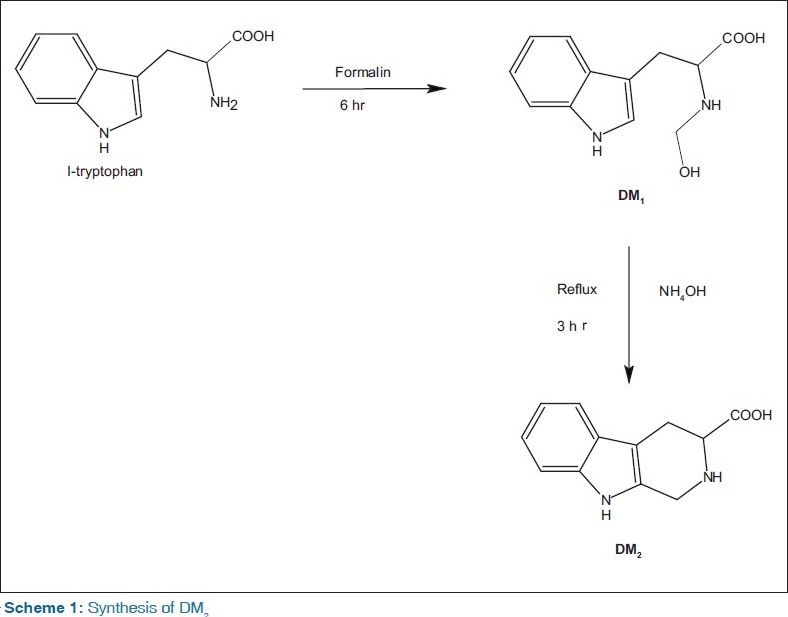
Synthesis of DM_2_

**Scheme 2:**
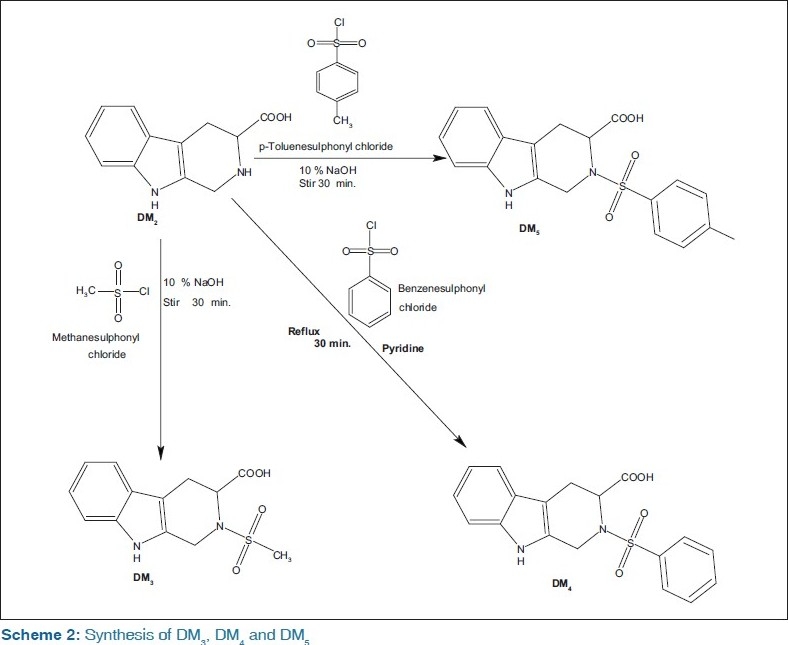
Synthesis of DM_3_, DM_4_ and DM_5_

The structures of synthesized compounds were confirmed by thin layer chromatography (TLC), m.p, IR, ^1^H-NMR, and mass spectrometry (MS) spectral analysis. The compounds (DM_3_–DM_5_) were synthesized by the treatment of *l*-tryptophan with formaldehyde which resulted in the formation of an intermediate α-hydroxymethylamino-α-3-indolylpropionic acid (DM_1_) which after refluxing with ammonia solution for 3 h resulted in cyclized product DM_2_ (Scheme 1). The compound DM_2_ on further treatment with methanesulfonyl chloride, benzenesulfonyl chloride, and toluenesulfonyl chloride yields different 1,2,3,4-tetrahydro-9*H*-pyrido[3,4-b]indole-3-carboxylic acid derivatives (Scheme 2). The yield was found to be in range of 70–80%. The title compounds were confirmed by IR spectral data showing characteristic bands at 3650-3300 cm ^-1^corresponding to OH stretching, sharp band at 1690 cm^-1^ indicated the presence of C=O group. Compounds (DM_3_–DM_5_) were confirmed by stretching at 1330 and 1120 cm ^-1^due to the presence of the SO_2_ group. Compounds DM_1_–DM_5_ was confirmed by ^1^H-NMR spectral analysis. The NMR proton singlet peak at δ 11 ppm and 3.70–3.80 revealed the presence of carboxylic acid and aromatic N–H groups. Further appearance of molecular ion peak M at *m/e* 294 (43.9%) 356 (43.9%), and 370 (44.2%) confirmed the structures of compounds (DM_3_–DM_4_). The compounds DM_1_ and DM_2_ do not significantly decrease blood glucose level in streptozotocin-induced diabetes. Compounds DM_3_ and DM_4_ exhibited less activity, whereas compound DM_5_ i.e., was found to possess better antidiabetic activity.

## CONCLUSIONS

The yield of all 1,2,3,4-tetrahydro-9H-pyrido[3,4-b]indole-3-carboxylic acid derivatives were found to be in the range 70-80%. The purity of compounds was ascertained by meting point and TLC. The assigned structures were further established by IR, ^1^H-NMR, and mass spectral studies. The antidiabetic activity of the synthesized compounds was screened using streptozotocin-induced diabetes in rats. Pioglitazone was used as a standard drug. Compounds DM_3_ [2-(methylsulfonyl)-1,2,3,4-tetrahydro-9*H*-pyrido[3,4-b]indole-3-carboxylic acid] and DM_4_ [2-(phenylsulfonyl)-1,2,3,4-tetrahydro-9*H*-pyrido [3,4-b]indole-3-carboxylic acid] showed moderate activity, whereas compound DM_5_ [2-(*p*-toluenesulfonyl)-1,2,3,4-tetrahydro-9H-pyrido [3,4-b]indole-3-carboxylic acid] exhibited the highest antidiabetic activity.

From this study, it may be concluded that the 1,2,3,4-tetrahydro-9*H*-pyrido [3,4-b]indole-3-carboxylic acid compounds can be potentially be developed into useful antidiabetic agents that can prompt future researchers to synthesize a series of 1,2,3,4-tetrahydro-9*H*-pyrido [3,4-b]indole-3-carboxylic acid derivatives containing a wide variety of substituents with the aim of obtaining novel compounds with enhanced activity.
